# Accidental Hypothermia-Induced J Wave Coupled With Giant R Wave Augmented by Premature Atrial Contraction: A Case Report

**DOI:** 10.7759/cureus.60644

**Published:** 2024-05-20

**Authors:** Koji Takahashi, Hiroe Morioka, Shigeki Uemura, Takafumi Okura, Katsuji Inoue

**Affiliations:** 1 Department of Cardiology, Yawatahama City General Hospital, Ehime, JPN; 2 Department of Community Emergency Medicine, Ehime University Graduate School of Medicine, Ehime, JPN

**Keywords:** standard 12-lead electrocardiogram, premature atrial contraction, osborn wave, j wave, giant r wave, accidental hypothermia

## Abstract

The 12-lead electrocardiographic findings in hypothermia include the presence of J waves; prolongation of the PR, QRS, and QT intervals; and atrial and ventricular dysrhythmias. Among these findings, the J wave, known as the Osborn wave, is considered pathognomonic. In 1953, the J wave was reported as a specific response to hypothermia in dogs, representing the current at the site of injury instead of a widening of the QRS complex that occurs caused by a conduction delay. The J wave is often accompanied by ventricular fibrillation. For the past 28 years, it was assumed that the hypothermia-induced J wave was mediated by the transient outward current. However, it was recently been reported that the J waves in some patients with hypothermia can be considered delayed conduction-related waveforms. Here, we present a case of hypothermia-induced J waves together with giant R waves, which have not been previously reported during hypothermia, augmented by short RR intervals arising from premature atrial contractions. Our observations indicate that the underlying mechanism for the genesis of J waves is indeed conduction delay and not transient outward currents.

## Introduction

The classic 12-lead electrocardiographic (ECG) findings in hypothermia include the presence of J waves; prolongation of the PR, QRS, and QT intervals; and atrial and ventricular dysrhythmias [1]. Among these, the J wave, also known as the Osborn wave, is the most striking feature and is considered pathognomonic for hypothermia [2,3]. The J point denotes the junction of the QRS complex and the ST segment on the ECG, marking the end of depolarization and beginning of repolarization. The J wave is a deflection with a dome or hump (camel's hump) morphology in the same direction as the R wave, immediately following the QRS complex [3].

In 1953, Osborn reported that a secondary wave (J wave) closely following an S wave as a response to hypothermia in dogs represented a current of injury rather than a widening of the QRS complex due to a conduction delay and that the ventricular fibrillation (VF) usually occurred at a rectal temperature below 25°C with conditions of a low arterial pH [4]. A strong inverse correlation is observed between the J wave amplitude and body temperature, and J waves are observed in 100%, 75%, and 11% of the patients with core temperatures <28.0°C, 28.0-31.9°C, and 32.0-34.9°C, respectively [5]. In the so-called J wave syndrome (JWS), including the Brugada syndrome (BrS) and early repolarization syndrome (ERS), a transmural voltage gradient occurs in the early phases of the action potential (phases 1 and 2). This is produced by a prominent transient outward current (I_to_)-mediated action potential notch in the ventricular epicardium but not the endocardium and is responsible for the inscription of the J wave on the ECG [3]. I_to_-mediated J waves are associated with the development of phase 2 reentry and life-threatening ventricular tachyarrhythmia [6]. Arrhythmias associated with JWS, hypothermia, and the acute phase of ST-segment elevation myocardial infarction are mechanistically linked to abnormalities in the manifestation of I_to_-mediated J waves [3]. On the other hand, ventricular tachyarrhythmias during hypothermia, which occur only in cases with J waves, do not increase mortality; however, once VF has occurred, it is often fatal [7]. The incidence of VF is unexpectedly low in hypothermic patients with J waves, varying from 0% to 2% [5]. The Osborn wave in hypothermia is considered benign [8]. Recently, it has been reported that the underlying mechanisms for the genesis of J waves can be differentiated from rate-dependent changes in the J waves. Tachycardia attenuates the J wave due to the I_to_-mediated differences in transmural repolarization and augments the J wave due to slower conduction from the endocardium to the epicardium during hypothermia [9].

Giant R waves, defined as a 50% or greater increase in R wave amplitude [10], develop particularly during myocardial ischemia [11,12]. However, this waveform has not been reported during hypothermia to date.

Herein, we describe a case of accidental hypothermia in which J waves were augmented by spontaneously documented premature atrial contractions. In addition to J waves, giant R waves were present, particularly in leads with J waves.

## Case presentation

A 55-year-old Japanese man, who had no apparent medical history but had been socially reclusive for nearly 40 years, was found unconscious in an unheated room in his home by a social worker in January 2024 and was transported by ambulance to our hospital. The patient had no family history of sudden death. On arrival, his core temperature was 28.1°C; systemic blood pressure, 103/76 mmHg; heart rate, 87 beats/min; and respiratory rate, 21 breaths/min, with an oxygen saturation in room air of 92%. The Japan Coma Scale score to indicate the patient's level of consciousness was 30; at this level, patients open their eyes in response to repeated calls with application of a pain stimulus (Table 1). No heart murmurs were audible upon auscultation; however, breathing sounds in the right lung were diminished. Venous blood tests revealed prerenal kidney failure due to dehydration (creatinine, 4.96 mg/dL; hemoglobin, 19.7 g/dL) resulting in metabolic acidosis (pH, 7.158; PCO_2_, 43.7 mmHg; bicarbonate, 15.2 mmol/L; anion gap, 32.3 mmol/L; lactic acid, 15.34 mmol/L), with a normal serum potassium level of 3.6 mEq/L (reference range: 3.6-5.0 mEq/L), a slightly elevated high-sensitivity cardiac troponin I level of 96.7 pg/mL (reference range: ≤18.4 pg/mL), a normal brain natriuretic peptide level of 10.3 pg/mL (reference range: ≤18.4 pg/mL), and a normal hemoglobin A1c level of 5.5% (reference range: 4.6-6.2 pg/mL). 

**Table 1:** Japan Coma Scale scoring Reference: [13]

Code	Score	Level of consciousness (for adults)
0-digit: alertness	0	Alert
1-digit: wakefulness without any stimuli	1	Almost fully conscious but not normal
	2	Unable to recognize time, place, or person
	3	Unable to recall name or date of birth
2-digits: arousable in response to some stimuli	10	Arousable by being spoken to
	20	Arousable by loud voice
	30	Arousable only by repeated mechanical stimuli
3-digits: coma	100	Unarousable but responds to avoid the stimuli
	200	Unarousable but responds with slight movements, including decerebrate or decorticate postures
	300	Does not respond at all

**Figure 1:**
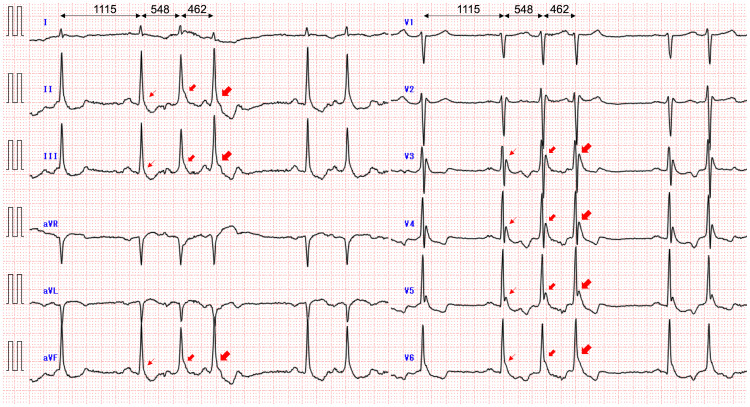
A 12-lead ECG obtained at a core body temperature of 28.1°C on admission Osborn waves, which are present particularly in the inferior and left precordial leads, are augmented by premature atrial contractions, whereas slurs remain slurs and notches remain notches. In addition, giant R waves in leads with J waves, vertical P-wave axis, rightward frontal QRS axis shift, and phasic voltage variation of QRS complexes when compared to those on the follow-up ECG are also shown. Numbers indicate RR intervals (ms). The arrows indicate Osborn waves. ECG: electrocardiogram

**Figure 2:**
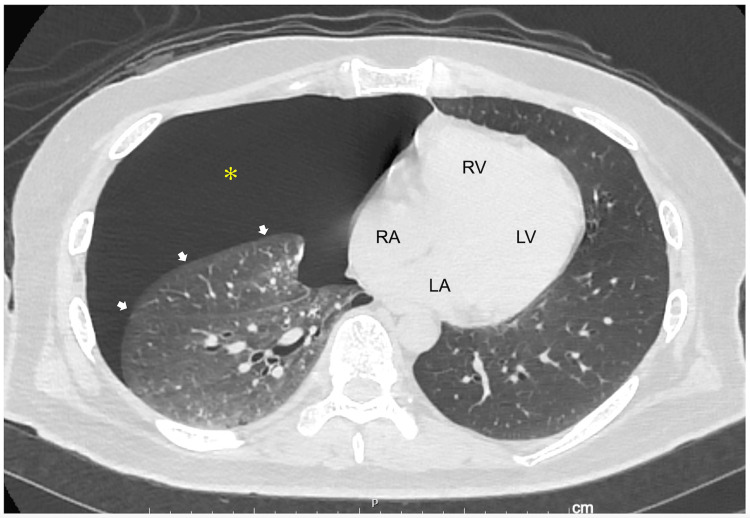
An axial computed tomography image of the chest on admission showing right pneumothorax A right pneumothorax (asterisk) with the build-up of air between the collapsed right lung (arrows) and chest wall is shown. The heart was suspected to develop a leftward shift. LA: left atrium; LV: left ventricle; RA: right atrium; RV: right ventricle

The admission ECG showed Osborn waves with augmentation by premature atrial contractions when J waves were defined as notches or slurs with an amplitude ≥0.1 mV above the isoelectric line in ≥2 contiguous leads, at the terminal part of the QRS complex [5,9,14], and augmentation (attenuation) of the J waves was defined as an increase (decrease) in amplitude by ≥0.05 mV [9,14] (Figure 1). In addition, the giant R waves, vertical P-wave axis, rightward frontal QRS axis shift, and phasic voltage variation of QRS complexes were also present when compared to those on the follow-up ECG. Chest computed tomography revealed a right pneumothorax (Figure 2). Echocardiography revealed no abnormal wall motion in the left or right ventricles.

**Figure 3:**
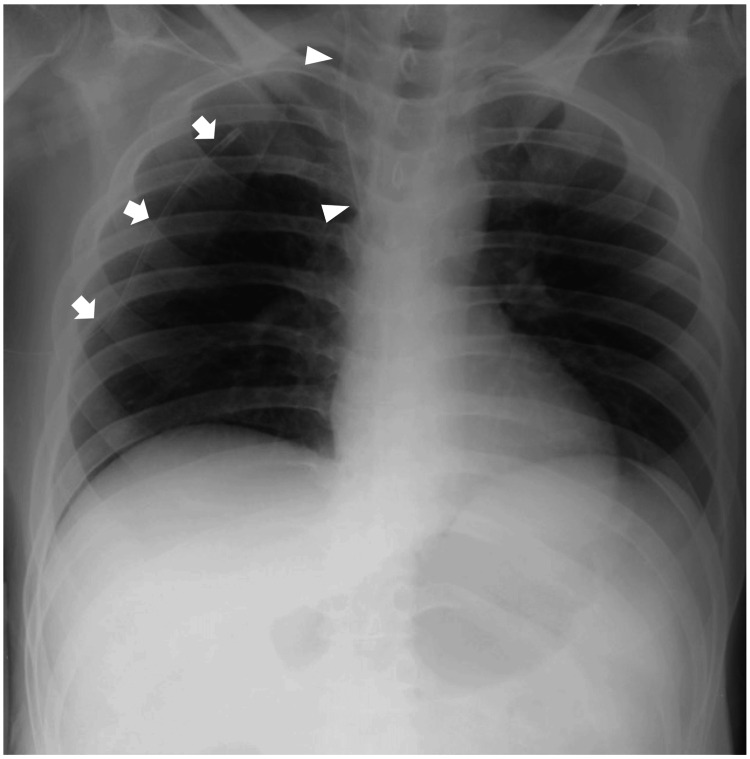
A plain film chest radiograph following chest drain insertion obtained on day 2 of hospitalization The right lung is re-expanded with no evidence of pneumothorax or overt pulmonary congestion. The cardiac silhouette is normal. The arrows indicate a chest drain appropriately positioned with its tip pointing superiorly within the pleural cavity. The arrowheads indicate a central venous catheter inserted via the right internal jugular vein, with its tip placed in the superior vena cava or at the cavoatrial junction.

The patient fully recovered without neurological impairment after rewarming treatment by using a heating blanket and intravenous administration of a sufficient volume of warmed fluids, although intravenous fluids, even when warmed, are not effective in rewarming. In addition, a chest drain was inserted using the standard technique, and the right lung was re-expanded with no complications such as re-expansion pulmonary edema (Figure 3). The serum creatinine level (0.93 mg/dL) and hemoglobin level (13.7 g/dL) reached normal limits.

**Figure 4:**
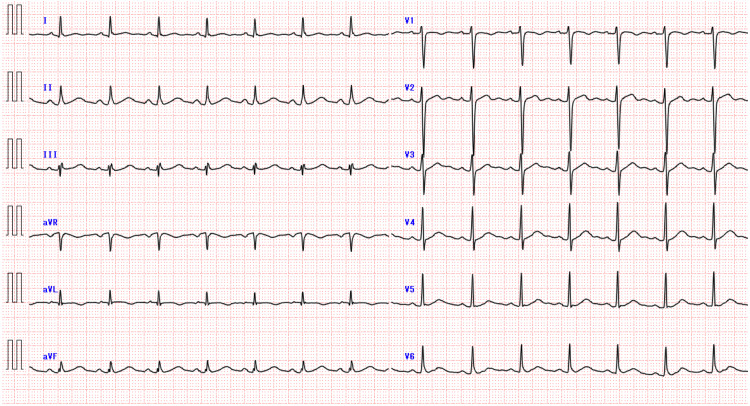
A 12-lead ECG obtained on day 6 of hospitalization The ECG shows the disappearance of prominent J waves, giant R waves, vertical P-wave axis, rightward frontal QRS axis shift, and phasic voltage variation in QRS complexes. RSR pattern in lead III and notched R in the ascending part of the R wave in lead aVF, which are considered fragmented QRS complex, in addition to the early repolarization pattern with notched or slurred J-point elevation ≥0.1 mV in leads I, II, and V6 are shown. ECG: electrocardiogram

Continuous ECG monitoring documented no ventricular tachyarrhythmias, and the follow-up ECG showed the disappearance of prominent J waves, vertical P-wave axis, rightward frontal QRS axis shift, phasic voltage variation of QRS complexes, and giant R waves. However, fragmented QRS complexes (fQRS) such as the RSR pattern and notched R in the ascending part of the R wave and early repolarization patterns were observed (Figure 4). The following tests were performed to determine if organic heart disease was present. Echocardiography revealed normal left and right ventricular function with a left ventricular ejection fraction of 67%, and contrast-enhanced cardiac magnetic resonance imaging showed no late gadolinium enhancement in the heart. Thus, fQRS and early repolarization patterns were considered as a benign ECG variant because the patient had no family history of sudden death, did not experience events due to ventricular tachyarrhythmias, and had normal echocardiographic and cardiac magnetic resonance images.

## Discussion

In our patient with hypothermia, the admission ECG showed both notched and slurred J waves, and the amplitude of these waves was augmented by premature atrial contractions. The J waves were considered as delayed conduction-related waveforms. In addition, giant R waves were present, particularly in the leads with J waves. The mechanism of these waves was also considered to be a conduction delay.

The J wave in the JWS as inherited ion channelopathies is considered I_to_-mediated [6], although some cases of BrS and ERS are considered subtle subepicardial cardiomyopathies and not primary electrical disorders [15]. Thus, factors that influence I_to_ kinetics can modify the manifestation of the J wave. For example, I_to_ is reduced by an increase in the heart rate owing to its slow recovery from inactivation, resulting in a decrease in the magnitude of the J wave. On the flip side of this phenomenon, the J wave exhibits a bradycardia/pause-dependent augmentation [3,16]. In addition, an increase in the net repolarizing current due to either a decrease in inward sodium (I_Na_) or L-type calcium currents (I_Ca,L_) or an increase in I_to_ accentuates the notch, leading to an augmentation of the J wave. Hypothermia is thought to accentuate the action potential notch owing to the difference in the temperature quotient (Q10) for the activation of I_to_ and I_Ca,L_. That is, the effect of cold temperatures slowing the activation kinetics of I_to_ is less than that of I_Ca,L_, resulting in an increase in the net repolarizing current and registers as an augmentation of the J wave on the ECG [2,16]. In addition to inducing a more prominent notch, hypothermia produces a markedly slow conduction velocity due to the reduced I_Na_ as a consequence of the slow recovery of the sodium channel from the inactivated state. The conduction delay from the endocardium to the epicardium together with the widening of the epicardial action potential notch can unmask a latent J wave by moving it out of the QRS complex [2,14].

Aizawa et al. reported the differentiation of the underlying mechanisms for the genesis of J waves from rate-dependent changes in J waves [9]. Tachycardia induced by atrial pacing attenuates J waves due to I_to_-mediated differences in transmural repolarization and augments J waves due to delayed conduction [9]. Recently, hypothermia-induced J waves concomitant with atrial fibrillation, which are found in approximately 20% of hypothermic patients [1], have been reported to be augmented just after short RR intervals, and a conduction delay induced by the suppression of sodium channels has been postulated as a possible mechanism [5,14]. In addition, Aizawa et al. reported that when a change in the amplitude of 0.05 mV or more is considered significant, two of nine patients with hypothermia-induced J waves complicated by atrial fibrillation or premature atrial contraction showed a bradycardia-dependent J wave augmentation as expected for I_to_-mediated J waves, whereas J waves were augmented after short RR intervals in the remaining seven patients as expected for depolarization abnormality [14]. Higuchi et al. also reported that when change in amplitude of 0.1 mV or more is considered significant, hypothermia-induced J waves concomitant with atrial fibrillation exhibited an augmentation of the J wave following a short RR interval in four of the eight patients, but not in the remaining four patients [5].

Tachycardia-dependent augmentation of the J wave amplitude, suggesting a mechanistic role of conduction delay, is also associated with an increased incidence of ventricular tachyarrhythmias [17]. Takahiro et al. reported a case of accidental hypothermia complicated by atrial fibrillation and VF, in which the J wave was augmented immediately after a short RR interval, and intravenous infusion of isoproterenol decreased the magnitude of the J wave and was effective in managing electrical storms [18]. Hypothermia is an important trigger of ventricular tachyarrhythmias, particularly ERS but not BrS. Thus, in patients with J waves mainly due to delayed conduction, and even in patients without J waves, other factors such as QT interval on ECG and serum potassium level should be carefully monitored and corrected [6,7]. Isoproterenol, a β-adrenergic agonist capable of augmenting I_Ca,L_, reduces the amplitude of the J wave and prevents arrhythmogenesis associated with JWS by opposing the increased outward current forces [6]. Drugs acting on the I_to_, I_Ca,L_, or I_Na_ may be useful for delineating the mechanisms underlying J waves [6]. However, hypothermic hearts may not respond to cardioactive drugs. Drug metabolism is slowed, leading to potentially toxic plasma drug concentrations [19]. Therefore, drugs should not be administered to patients without ventricular tachyarrhythmia. Continuous ECG monitoring during rewarming is warranted, particularly in patients with hypothermia-induced J wave. If ventricular tachyarrhythmia occurs, cardiopulmonary resuscitation should be performed according to guidelines.

Giant R waves have been documented in myocardial ischemia [11]. Particularly in variant angina with ST-segment elevation, R waves with increased height often appear in association with reduced depth or disappearance of S waves in leads exhibiting ST-segment elevation and reflect a conduction delay in the ischemic myocardium [12]. Giant R waves during hypothermia have not yet been reported. In our patient, who was suspected not to have acute coronary syndrome, giant R waves were observed, particularly in the leads with hypothermia-related J waves on admission ECG, and the mechanism for the genesis of the giant R waves was also considered to be conduction delay. ECG manifestations including a vertical P-wave axis, rightward frontal QRS axis shift, and phasic voltage variation of the QRS complexes were also observed in our patient. These ECG abnormalities are suspected to be attributable to changes in the anatomical position of the heart in the thoracic cavity resulting from the right-sided pneumothorax [20]. Therefore, it is not possible to determine whether the giant R wave was simply due to hypothermia, right pneumothorax, or both. The height of the giant R waves observed on the admission ECG of our patient indicated phasic voltage variation but necessarily depended on the preceding RR intervals, meaning that giant R waves might be both hypothermia-related and right-sided pneumothorax. However, Krenke et al. reported that the height of the R wave did not increase in either of the limbs or chest leads in 18 cases of right-sided pneumothorax [21].

## Conclusions

In this patient, slower conduction from the endocardium to the epicardium during hypothermia could generate J waves with augmentation by short RR interval due to premature atrial contractions, and the J waves could be labeled as "benign" waveforms because ventricular tachyarrhythmias did not develop. Hypothermia-induced J waves have a unique phenomenon in which the J wave height varies with the length of the preceding RR interval and their mechanism of occurrence differs; regardless of the mechanism, they are associated with VF, albeit at a very low frequency. Thus, continuous ECG monitoring during rewarming is warranted, particularly in patients with hypothermia-induced J wave. The occurrence of giant R waves during hypothermia requires further investigation.
